# Testicular cancer metastasis to the soft tissue: A case report and review of the literature

**DOI:** 10.1016/j.radcr.2021.04.004

**Published:** 2021-04-30

**Authors:** Matthew A. Crain, Dhairya A. Lakhani, Aneri B. Balar, Daniel Martin, Cara B Lombard, Thuan-Phuong Nguyen

**Affiliations:** a School of Medicine, West Virginia University, Morgantown, WV 26506, USA; b Department of Radiology, West Virginia University, Morgantown, WV 26506, USA; c Section of Molecular Imaging, Department of Radiology, West Virginia University, Morgantown, WV 26506, USA; d Section of Abdominal Imaging, Department of Radiology, West Virginia University, Morgantown, WV 26506, USA; e Section of Musculoskeletal Radiology, Department of Radiology, West Virginia University, Morgantown, WV 26506, USA

**Keywords:** Soft tissue metastasis, Testicular cancer, BEP, bleomycin etoposide platinum, CT, computed tomography, GCT, germ cell, MRI, magnetic resonance imaging, PET, positron emission tomography, TIP, taxol ifosfamide platinum

## Abstract

While germ cell testicular cancer is rare and only accounts for 1% of cancers in males, it is the most common solid malignancy among men between 14 and 44 years of age. Testicular cancer can be surgically excised by orchiectomy and is highly responsive to both chemotherapy and radiation therapy. Therefore, testicular tumors generally have the best cancer prognoses, especially since the majority are localized in the initial stage. However, long-term outcome depends on the potential for germ cell testicular cancer to metastasize, both proximal to the testicles and distally throughout the body. Germ cell testicular cancer metastasis to soft tissue, including the trunk, and extremities, appears to be exceedingly rare, as reflected in the extremely limited number of published cases (total of seven patients reported in literature). Vague symptomatology, delayed medical attention, and inconsistent treatment compliance may contribute to testicular soft tissue metastasis and underreporting of these tumors. Here, we report a case of metastatic non–seminomatous germ cell testicular cancer with a large necrotizing, ulcerative mass in the left Iliopsoas muscle and posterior abdominal wall.

## Background

While testicular cancer is rare and only accounts for 1% of cancers in males, it is the most common solid malignancy among men between 14 and 44 years of age [1,2]. Most common testicular cancers are germ cell tumors (GCT). The sex cord stromal and other non–germ cell tumors are exceedingly rare [3].

Testicular GCT can be surgically excised by orchiectomy and are highly responsive to both chemotherapy and radiation therapy [1], [2], [3]. Therefore, testicular tumors generally have the best cancer prognoses, especially since the majority are localized, in the initial stage [3]. However, long-term outcome depends on the potential for GCT to metastasize, both proximate to the testicles as well distally throughout the body [2,3]. Testicular GCT tend to metastasize via the lymphatic system from the testicles to the retroperitoneal lymph nodes [4]. In more advanced cases, metastatic testicular cancer can progress to the lymph nodes in the neck, chest, and pelvis. The most common extra nodal sites of metastasis are the lungs, bones, liver, and brain [5]. Since GCT are sensitive to radiation therapy and chemotherapy, treatment of these metastasized forms is often effective [2].

Testicular cancer metastasis to soft tissue, including the trunk, and extremities, appears to be exceedingly rare, as reflected in the extremely limited number of published cases [4], [5], [6], [7], [8]. A systematic review of the literature over the past 35 years found a total of seven cases of soft tissue metastasis from primary testicular cancer. Following the development and clinical implementation of computed tomography (CT) scans, Husband and Bellamy did an early study of 650 patients with primary testicular cancer and determined that only three cases subsequently developed metastases to soft tissue muscle tumors [4]. Two of their patients had left psoas muscle tumors, while one patient had left iliac and middle gluteal muscle masses. Around the same time, Hans, Lindner, and Webster published a report of a 43-year-old patient with a history of left radical orchiectomy and radiotherapy who presented six years later with a lump and vague discomfort in his upper right arm as well as numbness in his fingers [6]. The tumor was excised and determined to be malignant. Following a course of radiation therapy, there was no evidence of relapse over the next twelve years.

Plaza, et al. conducted an extensive study over a 30-year period of 118 patients diagnosed with metastatic soft tissues stemming from a wide variety of primary tumor sites [7]. Out of all of these cases, only 1 patient, a 54-year-old man was determined to have testicular cancer metastasis to a soft tissue mass in his thigh. Similarly, Bilici, et al. published a case report of a 41-year-old patient with a history of a left radical orchiectomy and subsequent lesions in his liver and brain who presented with a lump, but no pain, in his left thigh 7 years after the diagnosis of testicular cancer [5]. Magnetic resonance imaging (MRI) and biopsy determined a soft tissue malignant mass, which had metastasized from primary testicular cancer. Excision of the tumor eliminated his symptoms with no recurrence during 10-months’ follow-up. Finally, Degirmencioglu, et al, described a 62-year-old patient with a history of a right radical orchiectomy and lymph node resection who declined chemotherapy, and subsequently presented with pain, loss of motion, and swelling in his right leg 20 months’ later [8]. Positron emission tomography (PET and/or CT) revealed a large hypermetabolic lesion into both the muscular soft tissue and bone, and a biopsy confirmed the diagnosis of metastasis from the earlier testicular malignancy. Here we report a case of non–seminomatous germ cell testicular cancer metastasis to iliopsoas muscle.

## Case Report

**Fig. 1:**
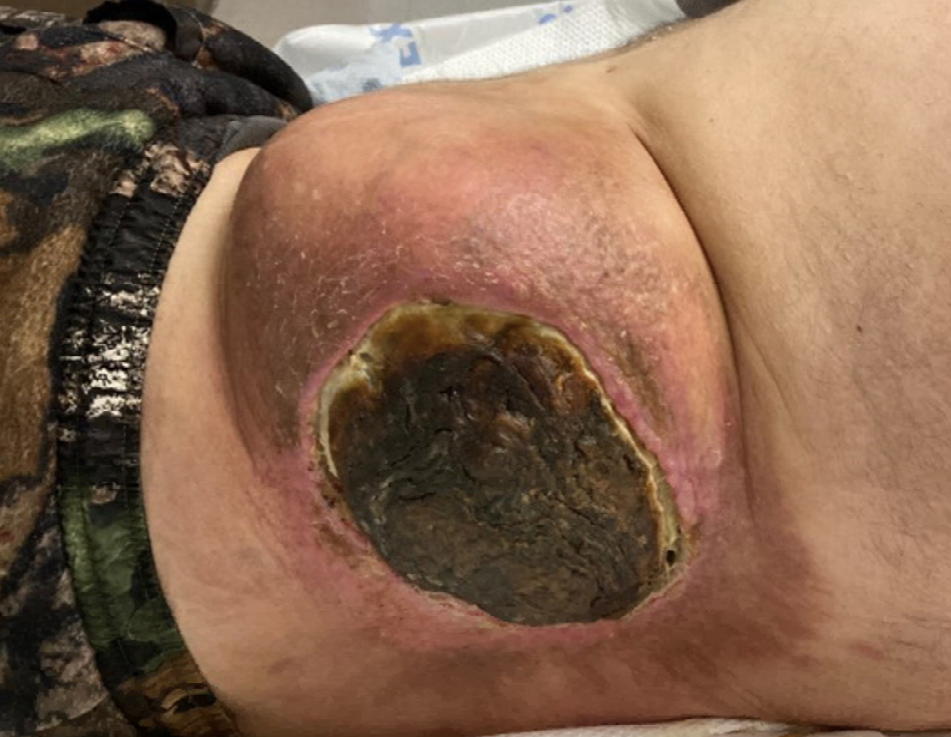
Clinical photograph illustrate a large necrotic left flank mass.

A 36-year-old, Caucasian male with prior history of left testicular cancer presented to our emergency department with a large left flank mass that had been necrotic and draining foul-smelling purulence for several months’ as well as severe pain from his left hip and flank (Fig. 1). His cancer history is extensive and notable.

**Fig. 2:**
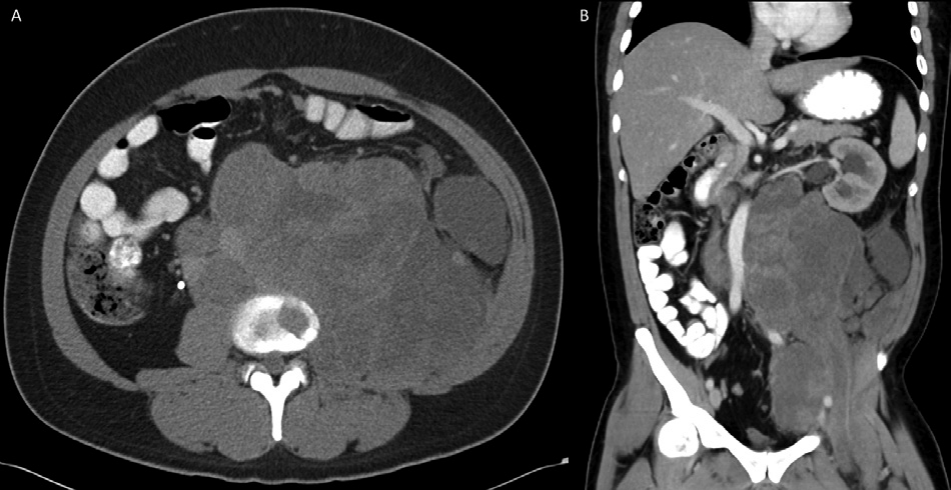
Axial (Fig. 1A) and coronal (Fig. 1B) computed tomography (CT) image of the abdomen 2 years following the initial diagnosis demonstrate a large heterogenous soft tissue mass arising from the left iliopsoas muscle. There was significant mass-effect on the abdominal viscera, with cranial displacement of the left kidney and right-ward deviation of the abdominal aorta.

**Fig. 3:**
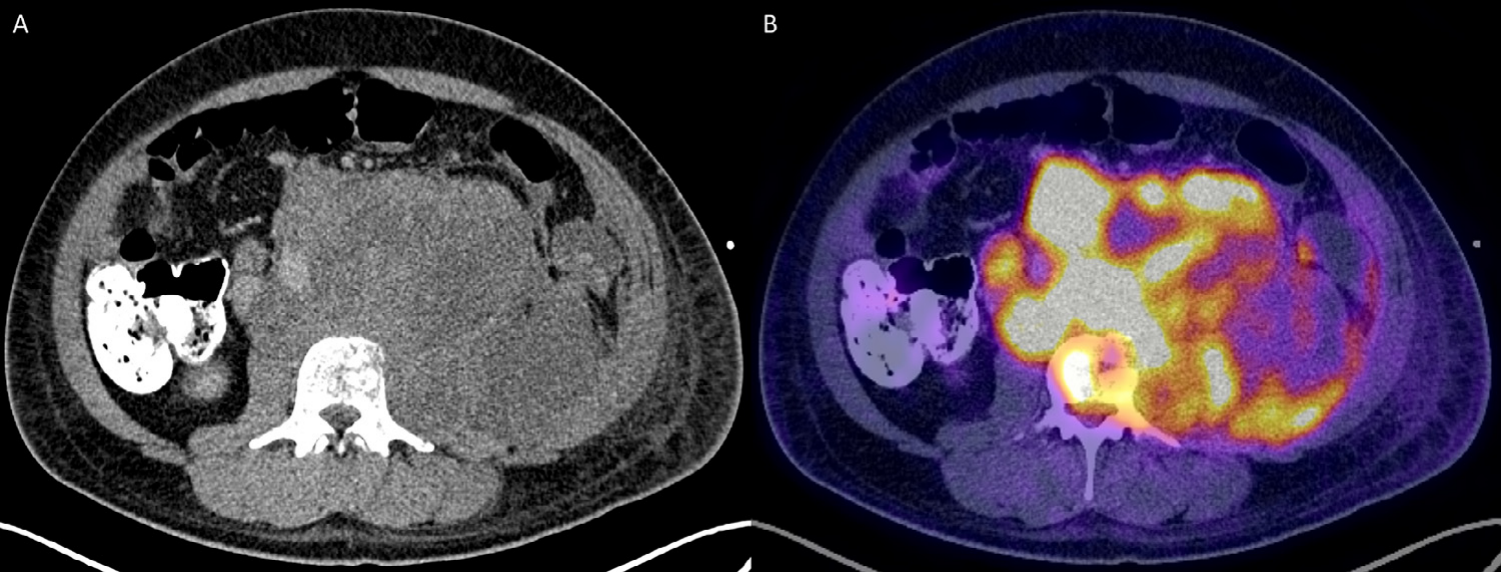
Positron emission tomography (PET) and/or computed tomography (CT) was subsequently performed. This mass exhibits peripheral, heterogenous increased FDG uptake, with extension into L1 through S1 vertebral bodies (not included in the figure).

The patient was initially incidentally diagnosed in 2010 with non–seminomatous germ cell testicular cancer with 95% embryonal and 5% yolk cell components, which was managed with a left inguinal total orchiectomy. The patient delayed recommended bleomycin, etoposide, and platinum (BEP) chemotherapy and radiation therapy following surgery. In 2012, he presented with obstructive uropathy, from external compression secondary to large retroperitoneal mass, requiring nephrostomy tube placement (Fig. 2A). This heterogenous soft tissue mass demonstrated internal necrosis and measured 19.6 × 17.8 × 2.6 cm, arising from the left iliopsoas muscle, pushing the aorta, and kidney (Fig. 2B). PET/CT was performed for further evaluation which revealed heterogenous mass with increased peripheral metabolic activity with central necrosis (Fig. 3A) in addition to lytic bone lesions and abnormal hypermetabolic activity in the L1 through S1 vertebrae (Fig. 3B).

**Fig. 4:**
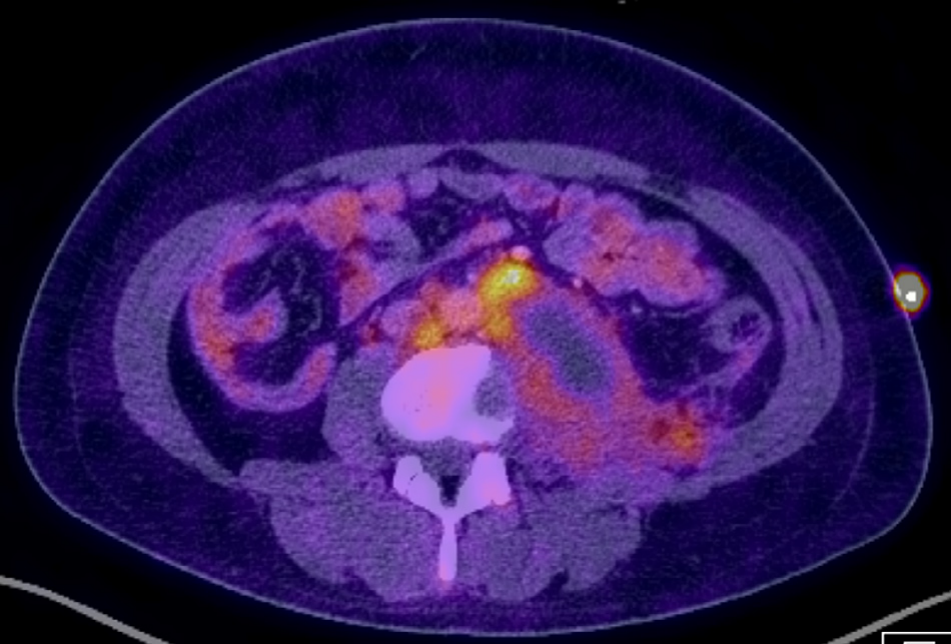
Follow-up Positron emission tomography (PET) and/or computed tomography (CT) image 3-months’ following treatment demonstrated decrease in size and metabolic activity of known mass, reflecting appropriate response to therapy.

CT guided biopsy of this lesion showed metastatic non–seminomatous germ cell tumor without embryonal carcinoma component. BEP chemotherapy was initiated, and follow-up PET and/or /CT three months’ later revealed decreased size and metabolic activity in this metastatic soft tissue mass (Fig. 4). In 2015, there was near-complete resolution of the disease and he was seen briefly for removal of his medication port.

**Fig. 5:**
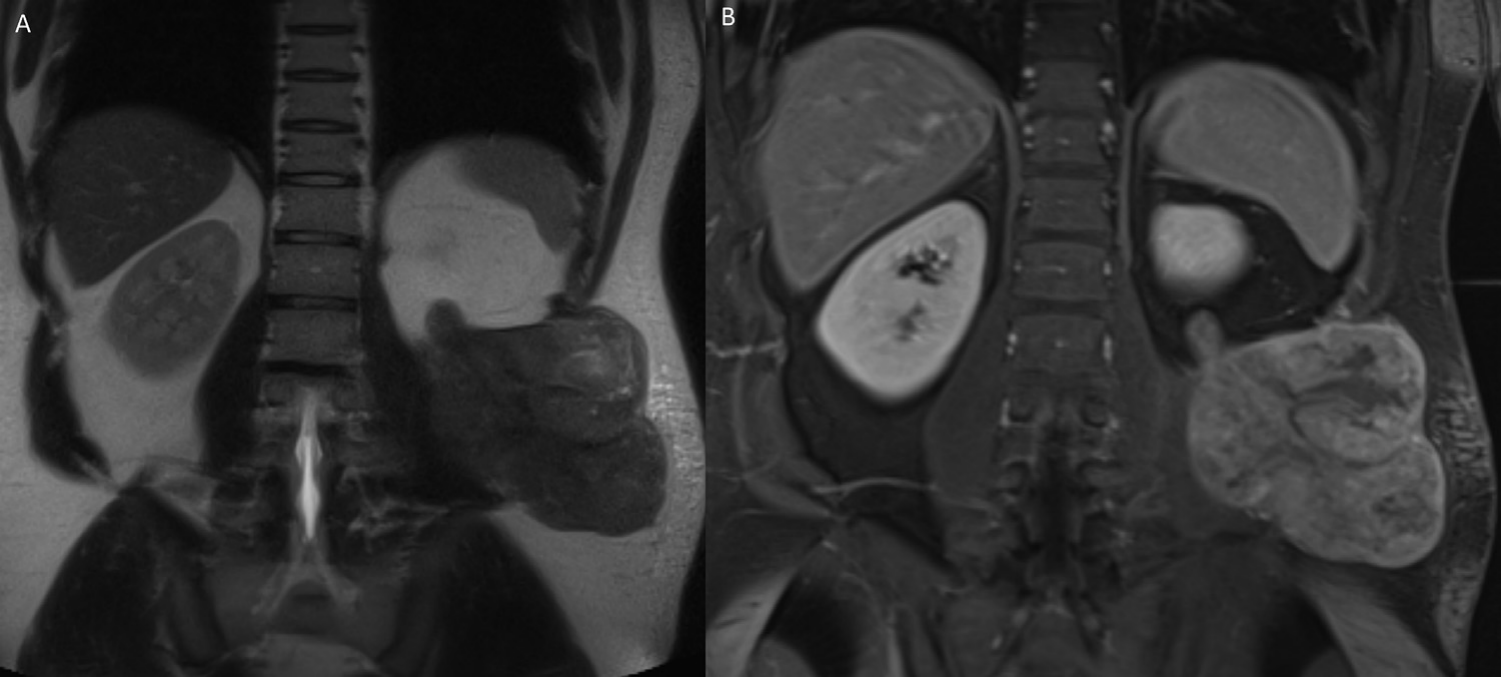
Two years following resolution of left iliopsoas mass, magnetic resonance imaging (MRI) of the abdomen, including T2 Half-Fourier Acquisition Single-Shot Turbo Spin Echo (HASTE) Coronal (Fig. 5A) and Postcontrast T1 Volumetric Interpolated Breath-hold Examination (VIBE) Coronal (Fig. 5B) images, was performed for diagnostic workup of new onset of left flank pain. This study showed a large, partially enhancing mass in the left retroperitoneal space, invading the left iliopsoas muscle and extends into the left flank soft tissues, reflecting recurrence of metastatic disease.

In 2017, the patient presented with uncontrollable constant left flank pain radiating into his back and groin. MRI of the abdomen was performed which revealed a large left flank mass measuring 11.0 × 8.0 × 11.0 cm, extending from the retroperitoneal space into subcutaneous soft tissue (Fig. 5). Biopsy confirmed the recurrence of a soft tissue mixed germ cell tumor. While he completed three cycles of taxol, ifosfamide, and platinum (TIP) chemotherapy as well as radiation therapy, the patient refused further management against medical advice and failed to follow-up, despite encouragement from his local physicians.

**Fig. 6:**
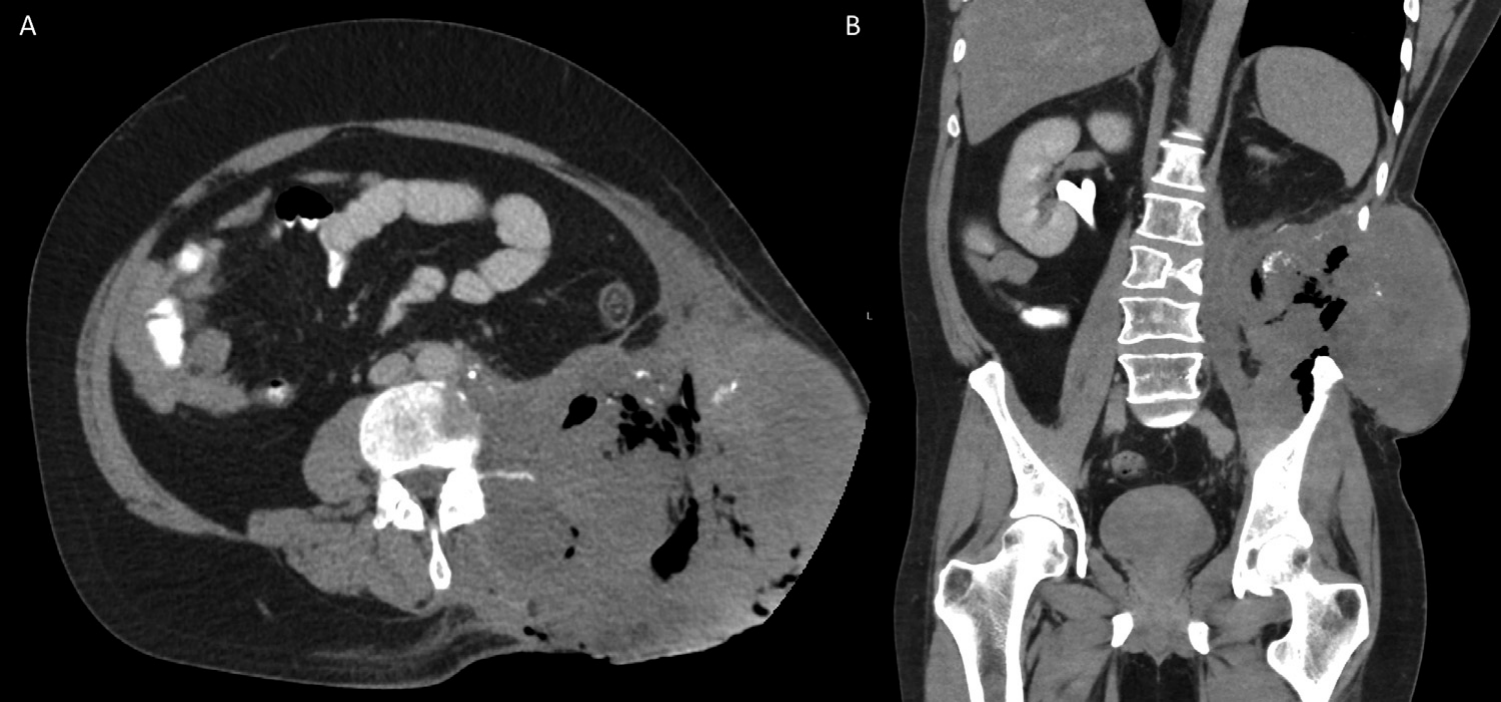
Four-years later, the patient presented with large ulcerative left flank mass (Fig. 1). Computed tomography (CT) of the abdomen and/or pelvis was performed for further evaluation, with axial (Fig. 6A), and coronal image (Fig. 6B). There was interval increase in size of the preexisting large necrotic left flank mass, which at this time had developed central necrosis with associated intramural air foci.

**Fig. 7:**
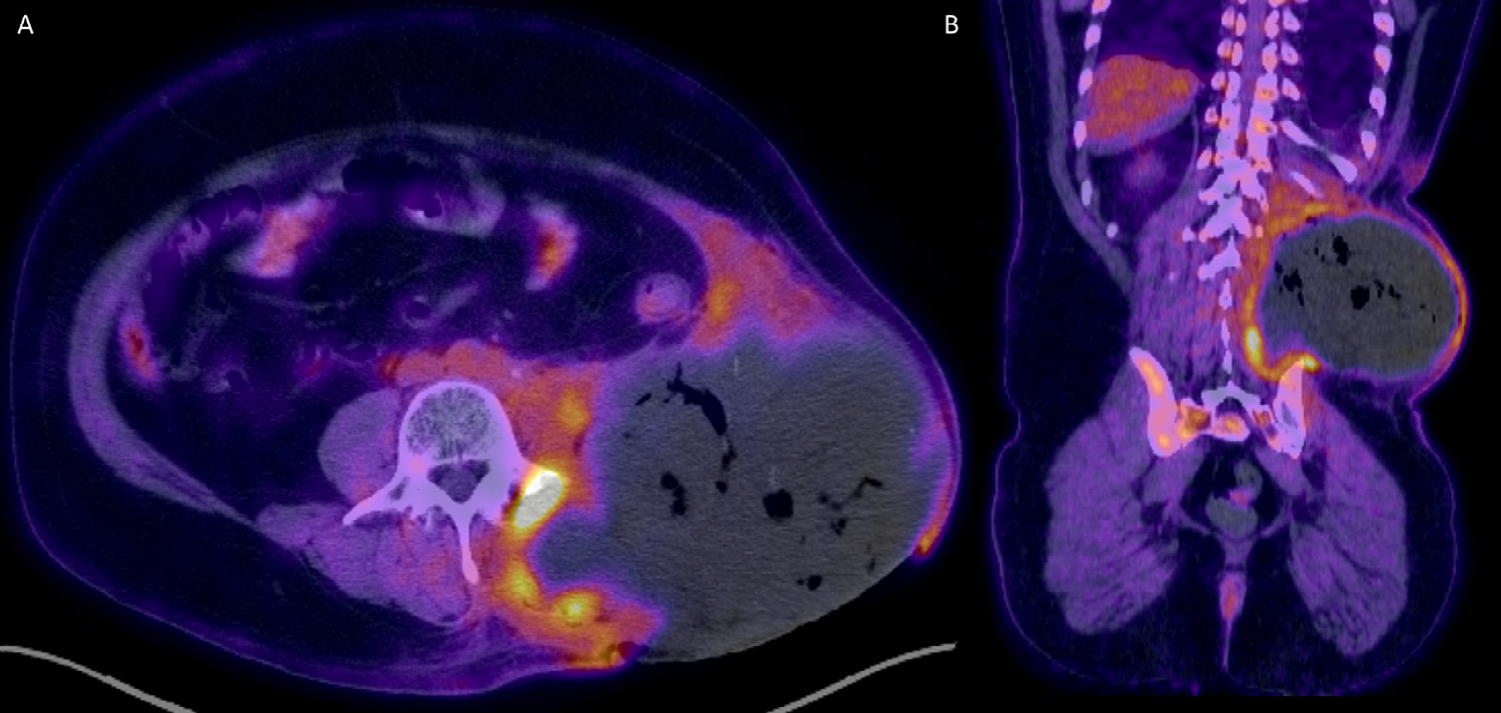
Subsequent evaluation with positron emission tomography (PET) / computed tomography (CT) showed peripheral areas of FDG uptake, compatible with malignancy.

When the patient finally returned to our hospital for this admission, his condition clearly had seriously deteriorated (Fig. 1). His local physician had recently prescribed a 3-months’ course of etoposide and platinum (EP) and Tecentriq in addition to ongoing antibiotics, with no improvement. Upon arrival, a CT-abdomen/pelvis was obtained, redemonstrating a large necrotic left flank soft tissue mass with involvement of the paravertebral and iliopsoas muscles, invasion of the posterolateral abdominal wall in the retroperitoneum, and concern for bony involvement of the left iliac bone as well as L3 vertebral body (Fig. 6). This mass measured 17.3 × 14.5 × 14.7 cm, which was larger than seen on the prior PET/CT in 2017. PET/CT demonstrated areas of increased metabolic activity, concerning for metastasis with superimposed infection (Fig. 7).

The case was presented to hospital medical review boards. The decision was made not to pursue surgical resection at this time since this would require extensive surgery with minimal benefit, and may lead to a large chronic non–healing wound. Chemotherapy was initiated with the hope of shrinking the tumor prior to surgical resection. The patient will be strongly encouraged to continue the full course of chemotherapy, follow up with his local providers, and obtain regular assessments, including radiological imaging studies, regardless of his symptomatology.

## Discussion

The presenting case is rare documentation of soft tissue metastasis from a primary testicular germ cell tumor, and appears to be only one of eight patients reported in the literature during the past 35 years. Therefore, it is important for cases to be shared so that this unusual condition is not missed, and a deeper understanding of this pathological process is understood. All patients appear to follow a similar progression of metastasis. Despite surgical removal of the primary tumor by radical orchiectomy, at times followed by a course of chemotherapy and/or radiation therapy, a soft tissue mass developed in bodily sites distant from the groin, including the trunk, and extremities, often years later. The pathophysiological process underlying this metastasis needs further study in order to develop methods to interrupt this sequence, including more systematic therapy and/or lymphadenectomy.

Another pattern among these patients is that they often were not fully compliant with treatment recommendations regarding chemotherapy and/or radiation therapy, or regular follow-up with specialists, which may have made metastasis more likely and progressive. This is particularly unfortunate since GCT are relatively sensitive to chemotherapy and radiation therapy, especially at the early stages. On the other hand, surgical resection of soft tissue tumors, especially in the abdomen and pelvis, may be complicated due to the anatomic challenges [9]. Therefore, early detection and therapy is especially important with soft tissue metastatic tumors.

Why do these patients appear to delay medical attention to, and treatment for these metastasized soft tissue tumors? Clinical research suggests that patients with soft tissue tumors often fail to seek and follow-up with medical services in a timely way [10]. This delay may simply be due to the lack of awareness of growing tumors, especially without significant pain or discomfort [11] as is common in soft tissue tumors in the abdomen and pelvis [9]. Denial and delays may be particular problems with metastases of testicular cancer since it is most prevalent in young men, who tend to feel invulnerable and often do not seek or comply with medical treatment. They also may face greater financial and insurance challenges, which could impact access to medical services, as it appeared to be in the presenting case.

While only a handful of cases have been reported in the literature, soft tissue testicular metastasis may be underdiagnosed for several reasons. First, the connection between a current soft tissue mass and testicular cancer years earlier may not always be made. Second, since soft tissue masses are generally underreported by patients, and soft tissue testicular metastases may be particularly ignored by young men, we may not have a complete clinical picture of this pathologic disease process. Further investigations are clearly needed.

There is a critical need to bring the attention of patients and healthcare providers to the importance of carefully and regularly observing symptoms following primary testicular cancer over the years. Patients need to be strongly encouraged to completely follow all treatment protocols, as well as routinely obtain comprehensive radiological imaging assessments, regardless of symptomatology, throughout their lifetime.

## Patient consent

Informed written consent was obtained from the patient.
